# Correction to: Functional characterization of genes mediating cell wall metabolism and responses to plant cell wall integrity impairment

**DOI:** 10.1186/s12870-019-1995-4

**Published:** 2019-09-05

**Authors:** Timo Engelsdorf, Lars Kjaer, Nora Gigli-Bisceglia, Lauri Vaahtera, Stefan Bauer, Eva Miedes, Alexandra Wormit, Lucinda James, Issariya Chairam, Antonio Molina, Thorsten Hamann

**Affiliations:** 1 Institute for Biology, Faculty of Natural Sciences, Norwegian University of Science and Technology, 5 Høgskoleringen, 7491 Trondheim, Norway; 2 Present Address: Division of Plant Physiology, Department of Biology, Philipps University of Marburg, 35043 Marburg, Germany; 3 Division of Cell and Molecular Biology, Department of Life Sciences, Imperial College London, Sir Alexander Fleming Building, South Kensington Campus, London, SW72AZ UK; 4 Present Address: Sjælland erhvervsakademi, Breddahlsgade 1b, 4200 Slagelse, Zealand Denmark; 5 Present Address: Laboratory of Plant Physiology, Wageningen University and Research, Wageningen, 6708PB The Netherlands; 6 Energy Biosciences Institute, University of California, 120A Energy Biosciences Building, 2151 Berkeley Way, MC 5230, Berkeley, CA 94720-5230 USA; 7 Present Address: Zymergen, Inc, 5980 Horton St, Suite 105, Emeryville, CA 94608 USA; 8 Centro de Biotecnología y Genómica de Plantas, Universidad Politécnica de Madrid (UPM)-Instituto Nacional de Investigación y Tecnología Agraria y Alimentaria (INIA), Campus de Montegancedo- UPM, Pozuelo de Alarcón, 28223 Madrid, Spain; 9 Departamento de Biotecnología-Biología Vegetal, Escuela Técnica Superior de Ingeniería Agronómica, Alimentaria y de Biosistemas, Universidad Politécnica de Madrid (UPM), 28040 Madrid, Spain; 10 Present Address: RWTH Aachen, Institute for Biology I, Worringerweg 3, 52056 Aachen, Germany; 11 Present Address: ADAS, Battlegate Road, Boxworth, Cambridge, CB23 4NN UK; 12 Present Address: Department of Nuclear Safety and Security, International Atomic Energy Agency, Vienna International Centre, PO Box 100, 1400 Vienna, Austria

**Correction to: BMC Plant Biol**

**https://doi.org/10.1186/s12870-019-1934-4**

Following publication of the original article [1], the author reported that the two curves in the sub-diagram WSR4 in Fig. 2a should be the other way round.

Correct Fig. 2 is as follows:

**Fig. 2:**
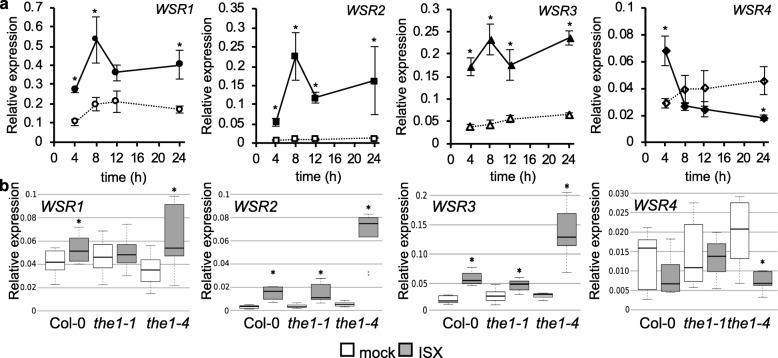
Candidate gene expression profiling in seedlings exposed to cellulose biosynthesis inhibition. **a** Gene expression of *WSR1, 2, 3* and *4* in Col-0 seedlings at the indicated time points after mock (DMSO; empty symbols, dotted lines) or ISX (filled symbols, solid lines) treatment according to qRT-PCR analysis. Values were normalized to *ACT2* and represent means from 3 independent experiments (one replicate per experiment with multiple seedlings pooled for each replicate). Error bars indicate SD. Asterisks indicate statistically significant differences (*p < 0.05) to mock controls according to Student’s t test. **b** Transcript levels of *WSR1, 2, 3* and *4* in Col-0, *the1–1* and the1–4 seedlings mock (DMSO) or ISX-treated for 8 h. Values were normalized to *ACT2* and represent means from 3 independent experiments (*n* = 8–9). Asterisks indicate statistically significant differences to mock controls according to Student’s t test (*p < 0.05). The boxes in the boxplot indicate interquartile range (IQR, between 25th and 75th percentile) and the black line in the middle of the box marks the median. The whiskers indicate data points furthest from the median, if they are still within 1.5xIQR from the closest quartile. The data points outside this range are plotted individually
