# Supplementary material for: Functional characterization of genes mediating cell wall metabolism and responses to plant cell wall integrity impairment
Source: BMC Plant Biol. 2019 Jul 18;19:320. doi: 10.1186/s12870-019-1934-4 (PMC6637594; doi:10.1186/s12870-019-1934-4)
Supplement: Supplementary file 1 — Table S1. Overview of genes selected for FTIR analysis. Table S2. DNA microarray-based expression analysis of 23 candidate genes in a published dataset (Hamann et al., 2009). Figure S1. Graphic representation of the transcript levels for 23 candidate genes in different cell types and plant organs based on data from the Genevestigator database. Figure S2. Graphic representation of average FTIR spectra of wild-type (Col-0) and all insertion lines screened. (a-c) Difference spectra obtained by subtraction from the Col-0 average spectrum. Black lines indicate 2x SD of Col-0-derived seedling material. Differently coloured lines indicate data derived from the insertion lines analysed. Genes where insertion lines exhibit similar FTIR-phenotypes are arranged together in panels (a-c). (d) Col-0 average spectrum. Figure S3. Characterization of WSR T-DNA insertion lines. Transcript levels of (a) WSR1, (b) WSR2, (c) WSR3 and (d) WSR4 were determined by qRT-PCR in wsrmutant seedlings. Values were normalized to ACT2 and represent means from 3 independent experiments (n.d.: not detectable). Error bars indicate SD, asterisks indicate statistically significant differences to Col-0 according to Student‘s t test (*p < 0.05; **p < 0,01; ***p < 0.001). Gene models indicating positions of T-DNA insertion (triangles) and PCR primers (arrows) are shown below bar charts. Exons are represented by boxes, introns by lines and untranslated regions by dashed lines. Figure S4. ISX-induced lignification in root tips of candidate mutants. Seedlings were mock or ISX-treated for 24 h. Representative images of phloroglucinol-stained roots are shown. Figure S5. Growth phenotypes of Col-0 and wsr plants at two time points. Material for cell wall analysis was harvested after 35 days. Figure S6. Lignin content in stems of adult wsr plants. Acetyl bromide soluble lignin was determined in stem cell wall preparations of 5 weeks-old plants. Bars represent mean values while error bars indicate SD (n = 4). (PDF [file 12870_2019_1934_MOESM1_ESM.pdf]

Table S1: 23 candidate genes were selected for FTIR analysis based on gene expression profiling (Hamann et al., 2009 and Genevestigator). Gene annotations are based on Araport11. T-DNA insertion lines used for FTIR spectroscopy-based screening and functional characterization are listed. Genes in bold were selected for further follow up studies.

AGI	Affymetrix Probe Set	Gene Annotation	T-DNA Insertion Lines
At1g07260	256053_at	UDP-GLUCOSYL TRANSFERASE 71C3	SALK_021979
At1g74440	260211_at	ER membrane protein, putative (DUF962)	SALK_059087
At2g02950	266745_at	PHYTOCHROME KINASE SUBSTRATE 1	SALK_005340
At2g13790	264107_s_at	SOMATIC EMBRYOGENESIS RECEPTOR-LIKE KINASE 4, BAK1-LIKE 1	SALK_057955
At2g20010	265583_at	Gls protein (DUF810)	SALK_004645
At2g35730	265796_at	Heavy metal transport / detoxification superfamily protein	SALK_058271 (*wsr2-1*)<br>SALK_123509 (*wsr2-2*)
At2g41820	260494_at	PXY/TDR-CORRELATED 3, Leucine-rich repeat protein kinase family protein	SALK_082484 (*wsr4-1*)<br>SALK_121365 (*wsr4-2*)
At3g09010	259213_at	Protein kinase superfamily protein	SALK_116262
At3g11340	256252_at	UDP-DEPENDENT GLYCOSYLTRANSFERASE 76B1	SAIL_1171_A11
At3g13650	256781_at	Disease resistance-responsive (dirigent-like protein) family protein	SALK_046217 (*wsr1-1*)<br>SALK_092919 (*wsr1-2*)
At3g16560	258437_at	Protein phosphatase 2C family protein	SALK_023206
At4g33300	253377_at	ADR1-LIKE 1	SAIL_302_C06
At4g33420	253332_at	PEROXIDASE 47	SM_3_37097
At4g35630	253162_at	PHOSPHOSERINE AMINOTRANSFERASE 1	SALK_074264
At5g24140	249773_at	SQUALENE MONOOXYGENASE 2	SALK_012094
At5g24430	249730_at	Calcium-dependent protein kinase (CDPK) family protein	SALK_028536
At5g40760	249372_at	GLUCOSE-6-PHOSPHATE DEHYDROGENASE 6	SALK_016157
At5g47730	248769_at	SFH19, Sec14p-like phosphatidylinositol transfer family protein	SALK_039575 (*wsr3-1*)<br>SALK_079548 (*wsr3-2*)
At5g48460	248656_at	ATFIM2	SALK_019403
At5g49360	248622_at	BETA-XYLOSIDASE 1	SALK_054483
At5g56540	247965_at	ARABINOGALACTAN PROTEIN 14	SALK_096806
At5g60660	247586_at	PLASMA MEMBRANE INTRINSIC PROTEIN 2;4	SM_3_20853
At5g65390	247189_at	ARABINOGALACTAN PROTEIN 7	SALK_039285

Table S2: DNA microarray-based expression analysis of 23 candidate genes in a published dataset (Hamann et al., 2009). Gene expression was monitored in 6 day-old mock- or ISX-treated Col-0 seedlings. Transcript levels are shown in affymetrix units after 0, 4, 8, 12, 18, 20, 24 and 36 hours of treatment.

AGI	Affymetrix	Mock								ISX							
	Probe Set	0h	4h	8h	12h	18h	20h	24h	36h	0h	4h	8h	12h	18h	20h	24h	36h
AT1G07260	256053_at	246,3	133,1	295,8	252,8	140,1	145,4	132,4	204,8	316,2	128,4	444,4	844,0	1360,3	1037,3	970,4	915,6
AT1G74440	260211_at	294,4	238,0	208,2	298,4	182,8	193,9	128,2	233,2	315,1	410,3	459,9	658,0	653,4	719,6	621,0	550,0
AT2G02950	266745_at	390,2	823,7	353,2	666,3	662,0	639,0	488,3	446,3	576,1	537,0	327,8	224,5	195,3	173,2	108,7	131,0
AT2G13790	264107_s_at	475,1	434,8	477,6	409,4	360,9	341,5	346,2	432,6	537,3	675,1	1579,3	1471,5	1433,6	1624,2	2058,9	1617,9
AT2G20010	265583_at	502,8	619,0	661,3	596,8	490,7	467,4	455,8	529,7	449,8	542,8	764,4	1055,4	1276,5	905,5	1476,9	1638,8
AT2G35730	265796_at	13,8	28,3	33,6	24,9	39,2	34,2	37,7	16,3	52,4	63,5	211,9	487,6	1257,1	925,2	1043,3	1521,3
AT2G41820	260494_at	303,0	327,1	291,5	364,8	409,6	300,5	413,7	291,1	265,6	243,0	121,2	62,8	53,5	40,3	27,8	32,4
AT3G09010	259213_at	123,9	130,3	71,6	134,2	115,2	124,3	88,7	178,7	111,3	227,3	462,4	673,5	891,7	820,4	777,8	1370,9
AT3G11340	256252_at	169,8	338,4	154,1	453,1	242,3	290,7	278,2	290,8	232,4	404,4	273,6	2105,1	3702,1	2350,8	2937,8	3559,5
AT3G13650	256781_at	597,2	603,9	638,8	573,2	590,6	792,4	384,7	789,9	720,9	685,6	292,7	1376,0	4018,1	2079,5	1931,3	3493,2
AT3G16560	258437_at	312,4	1164,6	315,3	1339,6	986,5	1167,4	958,0	1243,0	889,6	1051,6	306,2	204,4	170,8	168,0	134,5	190,6
AT4G33300	253377_at	682,8	500,3	657,5	853,5	646,3	555,2	472,8	766,4	469,4	998,1	2114,4	1814,2	1561,8	1970,4	2236,4	2219,1
AT4G33420	253332_at	759,8	598,0	910,3	688,8	714,0	691,1	429,5	617,5	593,8	587,8	1141,4	1763,0	1589,1	1462,7	1932,7	3207,4
AT4G35630	253162_at	3995,7	3516,6	3149,1	4586,4	3457,9	3259,3	3112,8	4025,1	4259,0	3840,5	6835,3	7991,2	8509,1	10097,4	10403,8	7576,9
AT5G24140	249773_at	62,5	88,9	65,0	45,3	47,9	80,6	100,4	96,4	22,9	99,1	226,8	93,4	243,2	319,5	307,5	203,1
AT5G24430	249730_at	377,4	322,1	447,6	342,3	309,8	295,1	324,0	319,2	367,3	431,7	894,9	1016,0	1295,8	1654,3	2177,9	1919,8
AT5G40760	249372_at	472,6	704,4	608,6	790,6	508,1	581,7	676,3	897,1	546,8	737,5	1110,7	1997,0	2801,2	2229,1	2563,4	3038,8
AT5G47730	248769_at	204,9	346,8	248,7	344,0	335,9	381,1	302,9	452,5	317,1	565,2	864,7	1047,1	1364,7	1223,2	1181,3	1461,5
AT5G48460	248656_at	199,3	261,8	232,1	281,0	318,9	341,3	315,0	275,7	284,3	256,8	202,4	75,7	97,8	128,9	97,9	49,8
AT5G49360	248622_at	1415,5	844,1	2865,0	1147,4	2686,0	2110,1	2905,3	2312,5	988,2	286,1	510,9	133,9	398,4	450,1	367,4	1013,9
AT5G56540	247965_at	1828,1	2143,6	2195,0	1727,8	1737,2	2042,2	1545,9	1777,0	1300,5	542,7	357,0	540,0	298,2	181,9	169,6	223,1
AT5G60660	247586_at	816,2	849,5	1122,4	854,7	856,2	1003,0	683,8	977,2	456,9	279,6	394,7	269,2	163,8	242,8	224,1	113,4
AT5G65390	247189_at	2453,3	2424,6	3070,6	2262,4	2899,1	2570,3	2765,7	1774,3	2618,2	1183,5	1203,7	844,1	761,1	866,5	1396,0	555,0

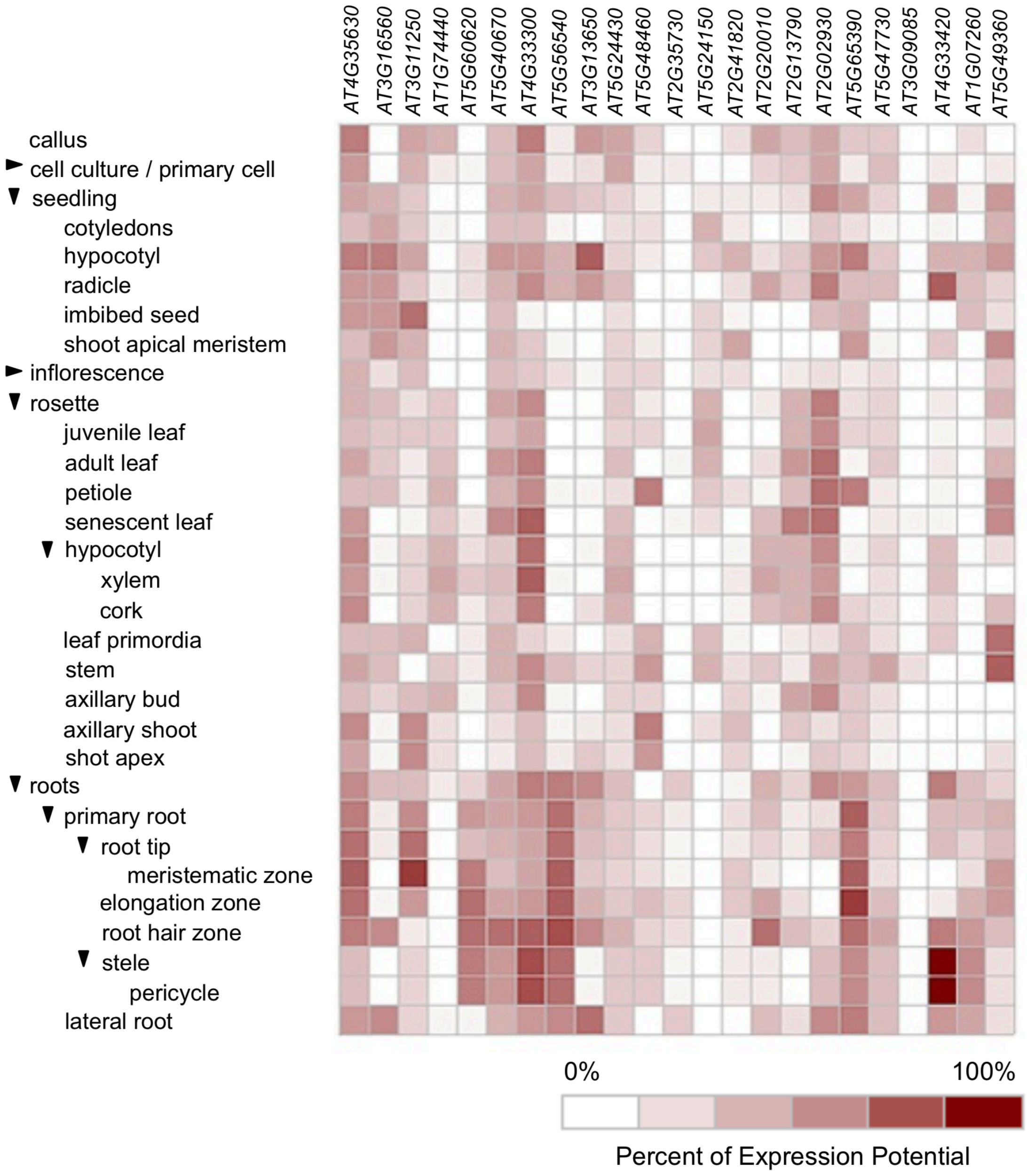

Figure S1: Graphic representation of the transcript levels for 23 candidate genes in different cell types and plant organs of *Arabidopsis thaliana* based on data deposited in the genevestigator database.

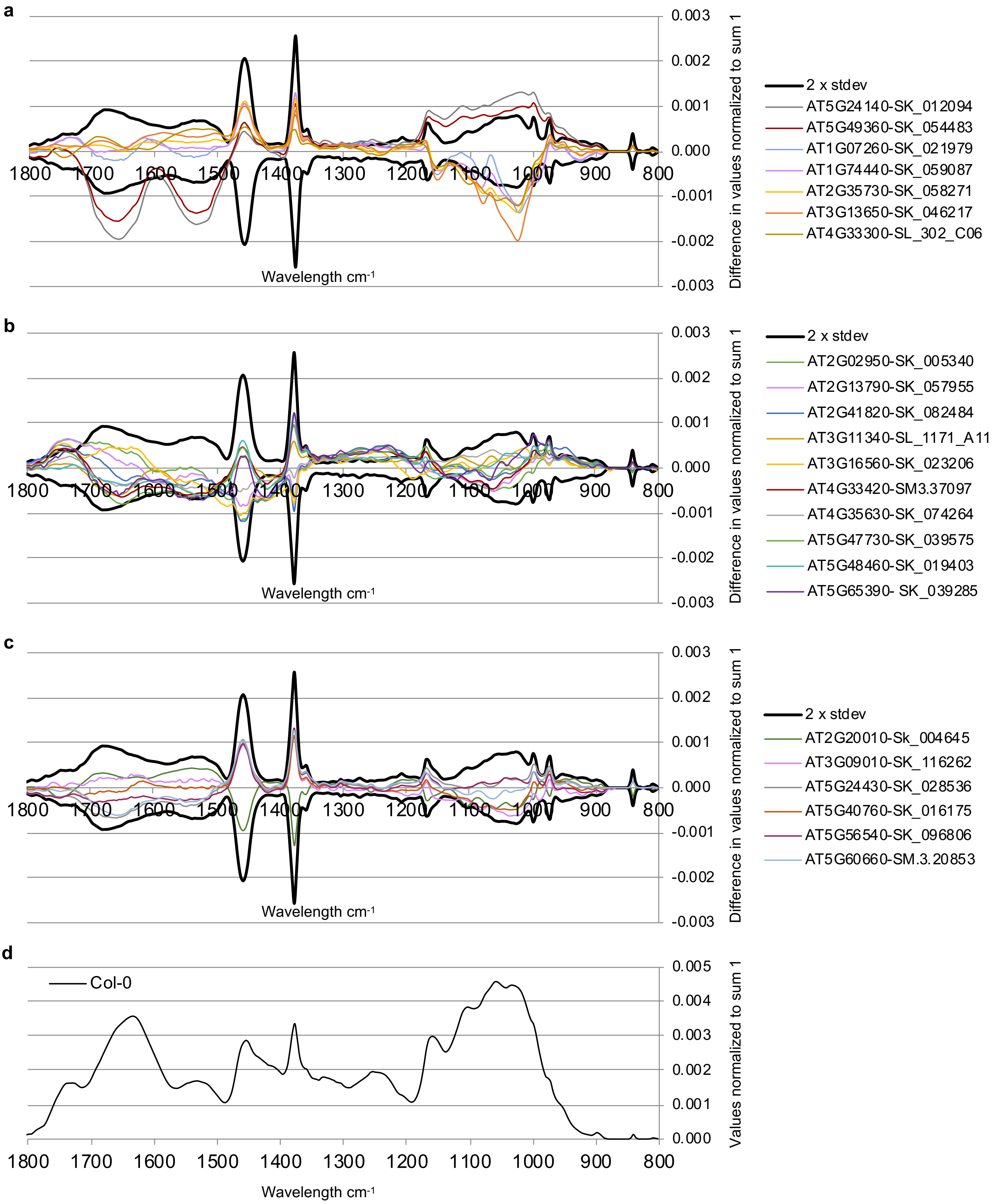

Figure S2. Graphic representation of average Fourier-Transform Infrared (FTIR) spectra of wild-type (Col-0) and all insertion lines screened. (**a-c**) Difference spectra obtained by subtraction from the Col-0 average spectrum. The black lines indicate 2x SD of the Col-0-derived seedling material. The different colored lines indicate data derived from the 23 insertion lines analysed. Genes where insertion lines exhibit similar FTIR-phenotypes are arranged together in panels (a-c). (**d**) Col-0 average spectrum.

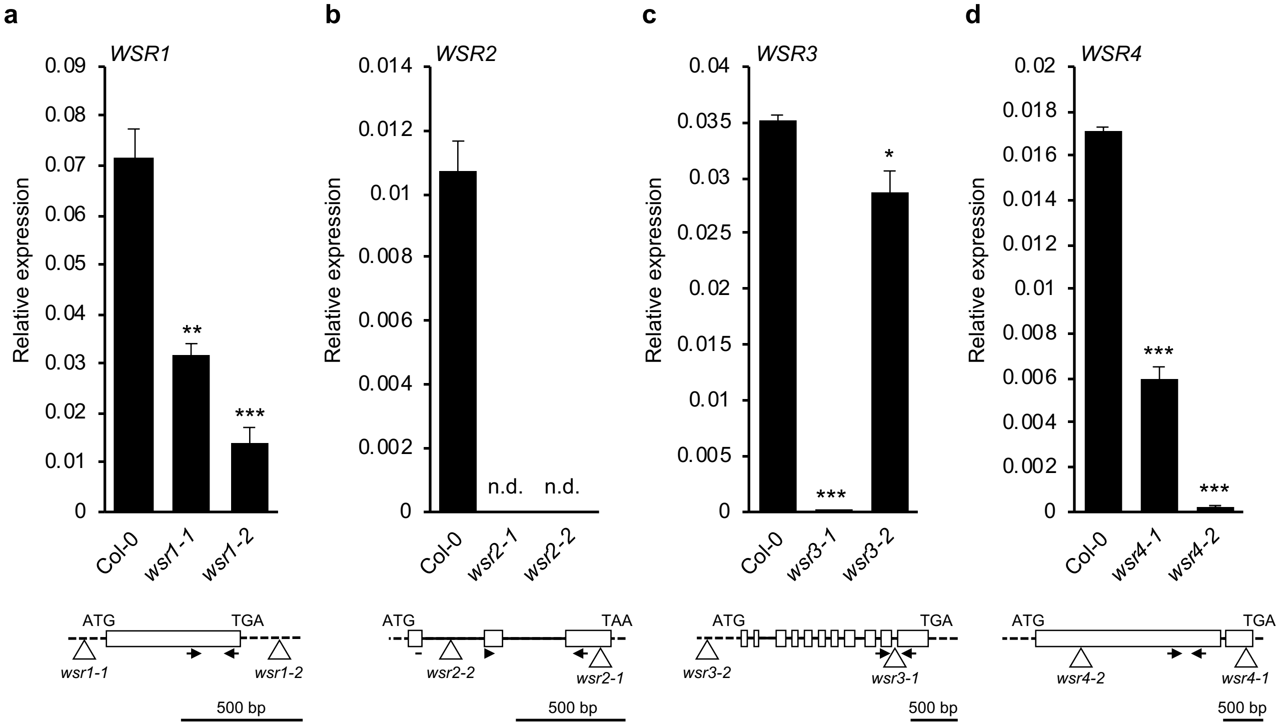

Figure S3: Characterization of *WSR* T-DNA insertion lines. Transcript levels of **(a)** *WSR1*, **(b)** *WSR2*, **(c)** *WSR3* and **(d)** *WSR4* were determined by qRT-PCR in the indicated *wsr* mutant seedlings. Values were normalized to *ACT2* and represent means from 3 independent experiments (n.d.: not detectable). Error bars indicate SD, asterisks indicate statistically significant differences to the wild type according to Student's t test (*p < 0.05; **p < 0,01; ***p < 0.001). Gene models indicating T-DNA insertion sites (triangles) and PCR primers used (arrows) are shown below the bar charts. Exons are represented by boxes, introns by lines and untranslated regions by dashed lines. Size bars indicate 500 bp.

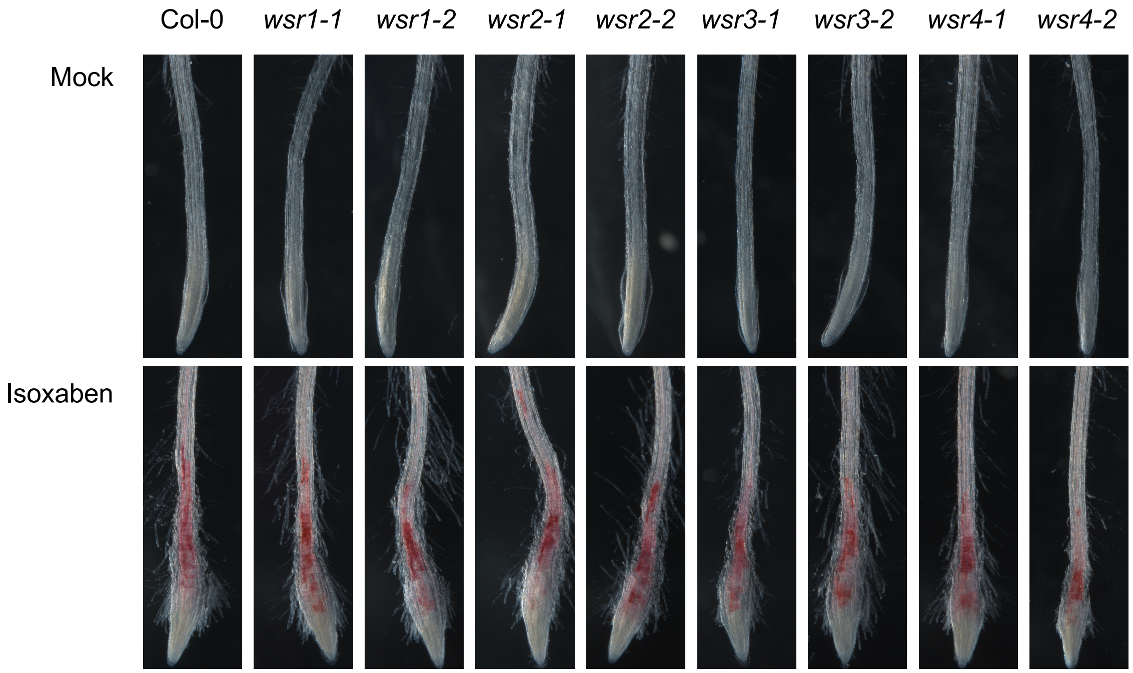

Figure S4: ISX-induced lignification in root tips of candidate mutants. Col-0, *wsr1-1*, *wsr1-2*, *wsr2-1*, *wsr2-2*, *wsr3-1, wsr3-2*, *wsr4-1* and *wsr4-2* seedlings were mock or ISX-treated for 24 h. Representative images of phloroglucinol-stained roots are shown.

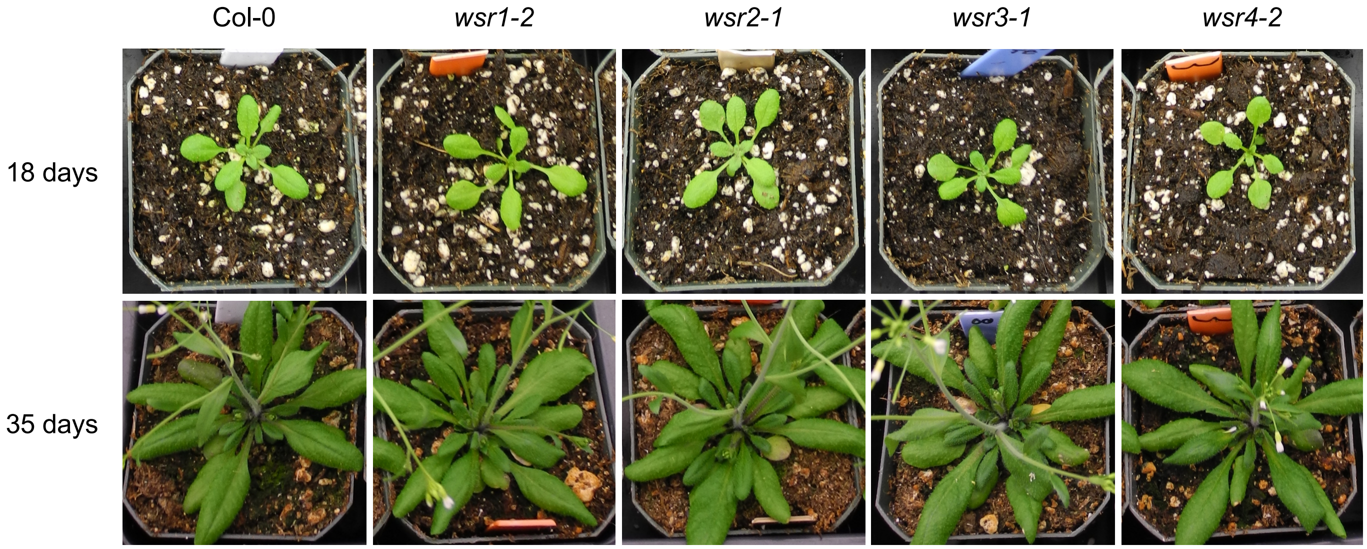

Figure S5: Growth phenotypes of Col-0 and *wsr* plants at two time points. Material for cell wall analysis was harvested after 35 days.

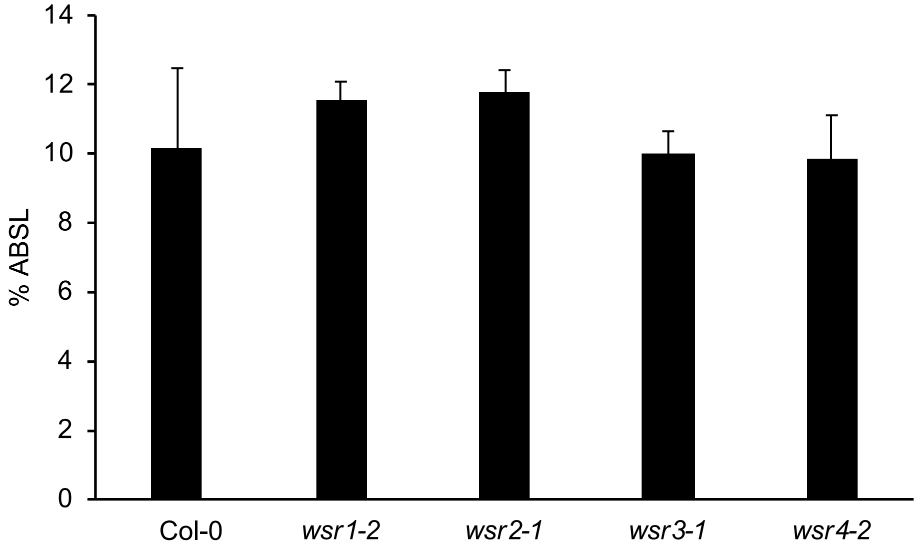

Figure S6: Lignin content in stems of adult *wsr* plants. Acetyl bromide soluble lignin was determined in stem cell wall preparations of 5 weeks-old Col-0, *wsr1-2*, *wsr2-1*, *wsr3-1* and *wsr4-2* plants. Bars represent mean values while error bars indicate SD (n= 4).
